# Surgical approach in type II Peters anomaly – case report

**DOI:** 10.22336/rjo.2022.20

**Published:** 2022

**Authors:** Cătălina-Ioana Tătaru, Călin Petru Tătaru, Laura Denisa Preoteasa

**Affiliations:** * Department of Ophthalmology, “Carol Davila” University of Medicine and Pharmacy, Bucharest, Romania; ** Department of Ophthalmology, Clinical Emergency Eye Hospital Bucharest, Romania

**Keywords:** Peters anomaly, corneal opacity, pediatric cataract, kerato-lenticular adhesions

## Abstract

**Objective:** The aim of this report was to present a rare case of apparently unilateral Peters anomaly and describe the clinical characteristics, surgical approach, and visual prognosis.

**Methods:** We presented the case of a 7-year-old female patient with posterior corneal defect due to kerato-lenticular adhesions along with anterior dislocation and opacification of the lens in the left eye and a history of post-traumatic evisceration of the right eye. Systemic associations included mental underdevelopment, left torticollis and scoliosis. No family history of acquired or inherited diseases were determined. We performed cataract extraction in the left eye and opted for aphakia.

**Results:** Based on clinical findings, we considered unilateral Peters anomaly type II. Cataract surgery slightly improved the visual acuity from hand moving to 20/ 400 UCVA (uncorrected visual acuity) and 20/ 100 with +10.0 diopters at 1 month postoperative. No enlargement of the corneal opacity was observed.

**Conclusions:** In this case, we were able to diagnose Peters anomaly only in one eye. The diagnosis required long follow-up with periodic measurement of intraocular pressure (IOP) to early detect glaucoma. The complexity and uniqueness of the case relied on the difficult approach made by the cloudy cornea and anterior lens dislocation. We applied a combination of techniques including adhesiolysis, cataract extraction and anterior vitrectomy. Further interventions such as secondary IOL (intraocular lens) implantation or PKP (penetrating keratoplasty) will be taken into consideration after six-month and one-year postoperative follow-up.

**Abbreviations:** PA = Peters anomaly, DM = Descemet’s membrane, IOL = intraocular lens, VA = visual acuity, OVDs = ophthalmic viscosurgical devices, IOP = intraocular pression, PKP = penetrating keratoplasty, BCVA = best corrected visual acuity, UCVA = uncorrected visual acuity

## Introduction

Peters anomaly (PA) is a very rare autosomal recessive congenital corneal opacity that is occasionally associated with systemic defects. It was first described in 1906 by Peters [**1**] in a patient with shallow anterior chamber, iridocorneal synechiae and central corneal leukoma. Nowadays, it is considered an anterior segment dysgenesis due to the incomplete separation of the cornea from the iris or the lens. 

PA is a rare disorder and its incidence was difficult to establish. Kurilec and Zaidman collected data from the Eye Bank Association of America, the Eye Bank for Sight Restoration in New York, the New York State Department of Health, and the Pediatric Keratoplasty Association and stated that approximately 1 infant corneal transplant is performed for every 24,000 live births and PA represents 60% of the causes [**2**]. In a previous study, Bermejo and Martinez-Frias found that 3.11 infants out of 100,00 births in Spain had congenital corneal opacity [**3**]. However, the incidence and prevalence of PA internationally remains unknown. 

Most cases are sporadic or recessive, but autosomal dominant inheritance has been reported [**4**]. Most common gene mutations include the deletion of PAX6 [**5**] and a single point mutation in FOXC1 [**6**], implicated in human anterior segment malformations. In 2006, Vincent et al. identified 4 mutations of CYP1B1, which may be responsible for 20% of cases with PA [**7**]. The literature review of PA of Bhandari et al. emphasizes how the mutation in these genes leads to abnormal cleavage of the lens vesicle from the surface ectoderm [**8**]. Embryologically, it is a mesenchymal dysgenesis of the ocular anterior segment, a spectrum of developmental disorders that include Axenfeld-Rieger syndrome, congenital glaucoma, posterior embryotoxon and sclerocornea [**4**]. 

Studies concerning the histopathology of PA illustrate immature or absent Descemet’s membrane (DM), thinning or absence of Bowman’s membrane, focal absence of endothelium and defects in the posterior stroma [**9**]. A new finding made by Wei Ni and associates identifies an abnormal thickness of DM with a “multiple-layer” structure and pigment tissue inside the layers [**10**]. 

Peters anomaly can be classified into two subtypes. The classic type I is usually unilateral and consists of a central stromal opacity and iridocorneal adhesions arising from the collarette and attaching to the periphery of the opacity [**11**]. The corneal opacity is either central or peripheral, without vascularization and endothelium and DM defects result in corneal edema. Type II is frequently bilateral, but often asymmetric, with denser corneal opacification and cornea-lenticular contact with juxtaposition of the lens or cataract [**12**]. Other ocular anomalies include glaucoma in 50% of cases, microcornea, cornea plana, sclerocornea, chorioretinal coloboma, iris coloboma, angle and iris dysgenesis, persistent hyperplastic primary vitreous, microphthalmia, optic nerve hypoplasia and foveal hypoplasia [**4**,**11**]. Systemic malformations are usually associated with type II and appear to arise from the maldevelopment of the neural crests: bone anomalies, delayed skeletal maturation, facial dysmorphism, ear anomalies and congenital heart defects [**4**,**11**]. Peters plus syndrome comprises anterior chamber defects in association with cleft lip/ palate, short stature, abnormal ears, and cognitive developmental delay [**13**]. 

The definite diagnosis is based on the association of clinical features with genetic testing. There is no standard treatment approval, yet numerous surgical techniques have been tried during the latest years, including: penetrating keratoplasty [**13**], cataract aspiration or lensectomy [**14**], optical iridectomy [**15**], selective endothelial removal [**16**], trabeculectomy and implant of glaucoma draining devices [**17**]. However, postoperative results do not lead to high hopes due to severe amblyopia and other ocular complications such as glaucoma or retinal detachment. 

## Materials and methods 

A 7-year-old female patient presented to our service for evaluation of a focal corneal opacity, photopsia and visual impairment in the left eye. Ocular medical history revealed a posttraumatic evisceration of the right eye. General examination indicated left torticollis, scoliosis, and neurological underdevelopment. No family history of acquired or inherited diseases were determined. 

**Fig. 1:**
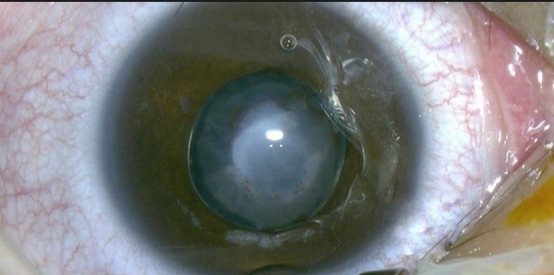
Left eye examination under general anesthesia - central corneal leukoma, anterior dislocated and opacified lens, corneo-lenticular adhesions

On clinical ophthalmic examination, visual acuity (VA) was hand motion in the left eye. BCVA (Best corrected visual acuity) and cycloplegic refraction could not be measured due to the postural abnormalities and lack of fixation. Examination of the affected eye under general anesthesia revealed a central leukoma with cornea-lenticular adhesions, anterior lens dislocation and opacification, and a bright red reflex (**Fig. 1**). 

**Fig. 2:**
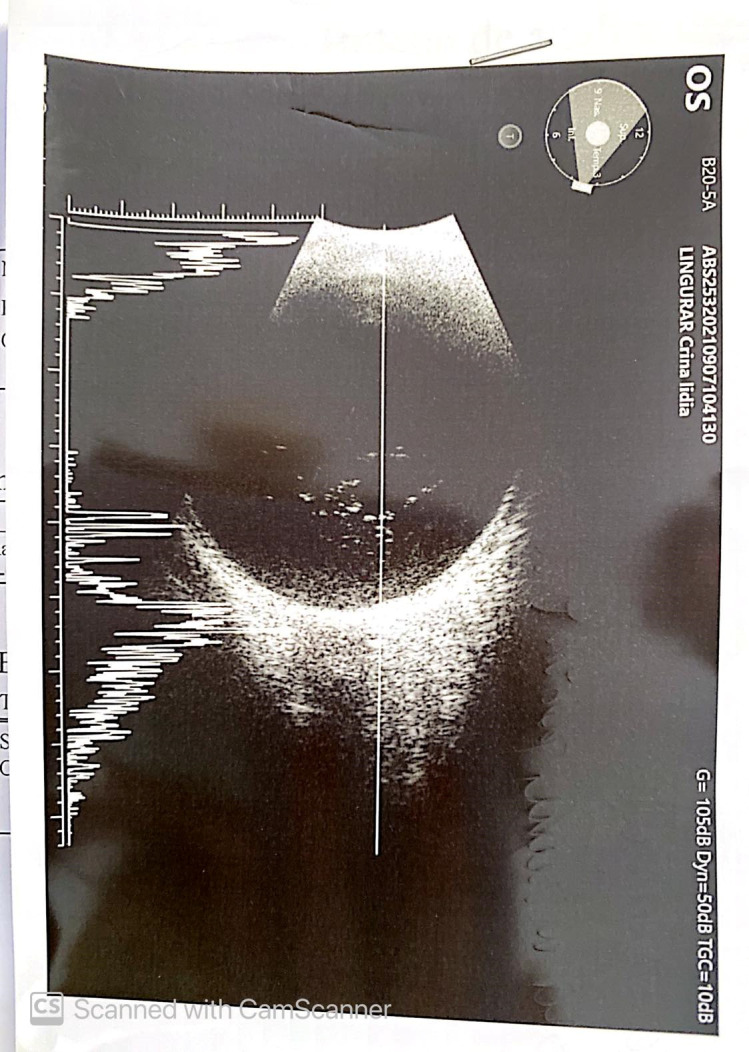
Conventional B-mode ultrasonography - Attached retina, vitreous degenerative echoes

The intraocular pressure was 17 mmHg measured with Maklakov applanation tonometer. Conventional B-mode ultrasonography showed an apparently healthy optic nerve and attached retina, with degenerative vitreous echoes (**Fig. 2**). 

**Fig. 3:**
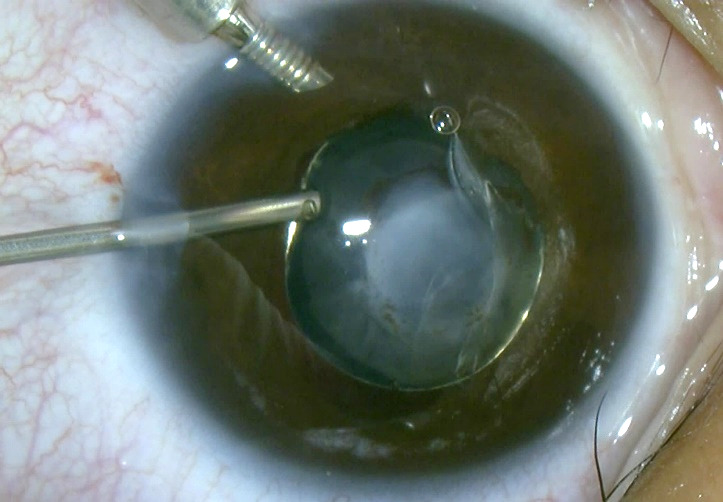
Making a breach in the anterior capsule with vitreous cutter

We decided to perform lens aspiration because the presence of cataract obliterated the visual axis and increased the risk of corneal decompensation due to kerato-lenticular adhesions. The procedure was performed by an experienced surgeon (C.P.T.) under general anesthesia. Two corneal incisions of 1.2 mm were made at 90◦ and 180◦ temporally. By using the maintainer, we assured the stability of the chamber. We made a breach in the anterior capsule at 8 o’clock with a 23-gauge vitreous cutter (**Fig. 3**) and performed hydro-dissection and hydro-delineation to create multiple cleavage plans within the lens.

**Fig. 4:**
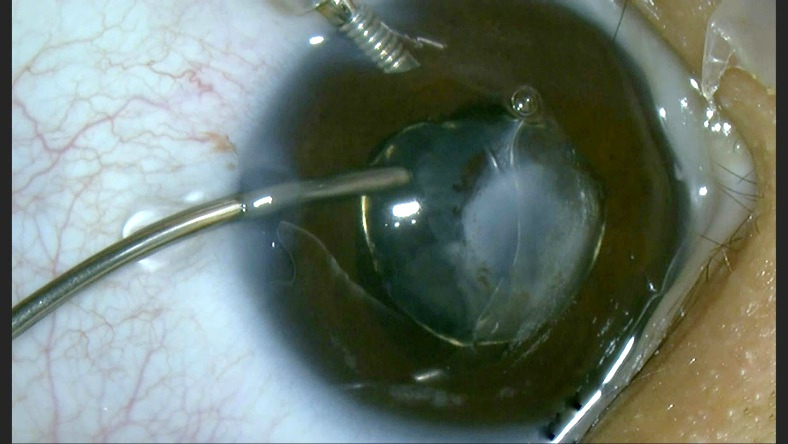
“In the bag” lens aspiration using manual irrigation-aspiration

Subsequently, we removed the masses with manual irrigation-aspiration within the capsular bag (**Fig. 4**). 

**Fig. 5:**
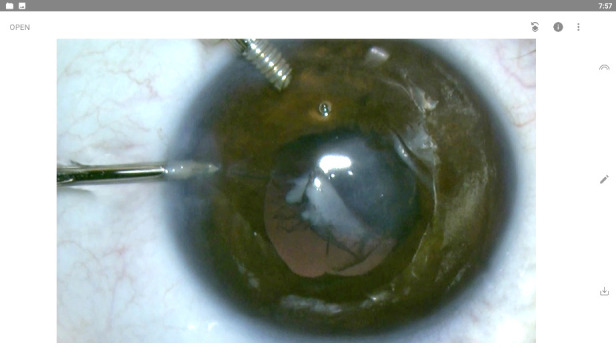
Adhesions’ removal using intraocular forceps

After extracting most of the lens material, we opted for vitrectomy to clean the remaining fragments. The capsular bag was also removed with the vitreous cutter. Limited anterior vitrectomy was performed to avoid vitreous tractions. When no masses were present in the anterior chamber, we were able to cut the adhesions between the cornea and the lens and gently extract them with an intraocular forceps (**Fig. 5**). 

**Fig. 6:**
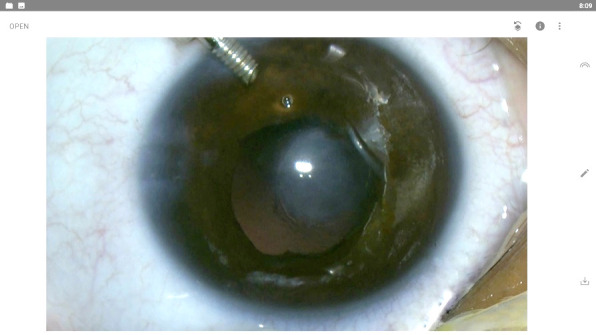
Postoperative aspect - central leukoma, aphakia, free peri-leukoma visual axis

At the end of the surgery, an air bubble was inserted to restore the anterior chamber and maintain its stability. The wounds were sealed using stromal hydration with balanced saline solution. A subconjunctival injection of dexamethasone was given at the end of the surgery (**Fig. 6**). 

Postoperative treatment consisted of topical antibiotics, corticosteroids, and anti-inflammatory drops 8 times daily tapering over 6 weeks. 

## Results

Based on clinical findings, an assessment of left Peters’ anomaly type II was made. Genetic testing was needed to confirm the diagnosis. 

The patient had an uncomplicated post-operative course with an improved visual acuity of 20/ 600 UCVA at 1 week. Due to a difficult communication, we could not realize hyperopia correction for the time being. Photopsia phenomenon was reduced, allowing slit lamp examination. No Descemet membrane detachment was diagnosed postoperatively, and the cornea appeared clearly, except for the previous leukoma. 

At 1 month postoperative, there was no progression of corneal opacity and UCVA increased at 20/ 400. We were not able to measure an exact refraction, however we attempted a correction of +10.0 diopters and obtained a corrected visual acuity of 20/ 100. The degree of amblyopia was not established, but we expected a moderate to severe level of visual impairment.

**Fig. 7:**
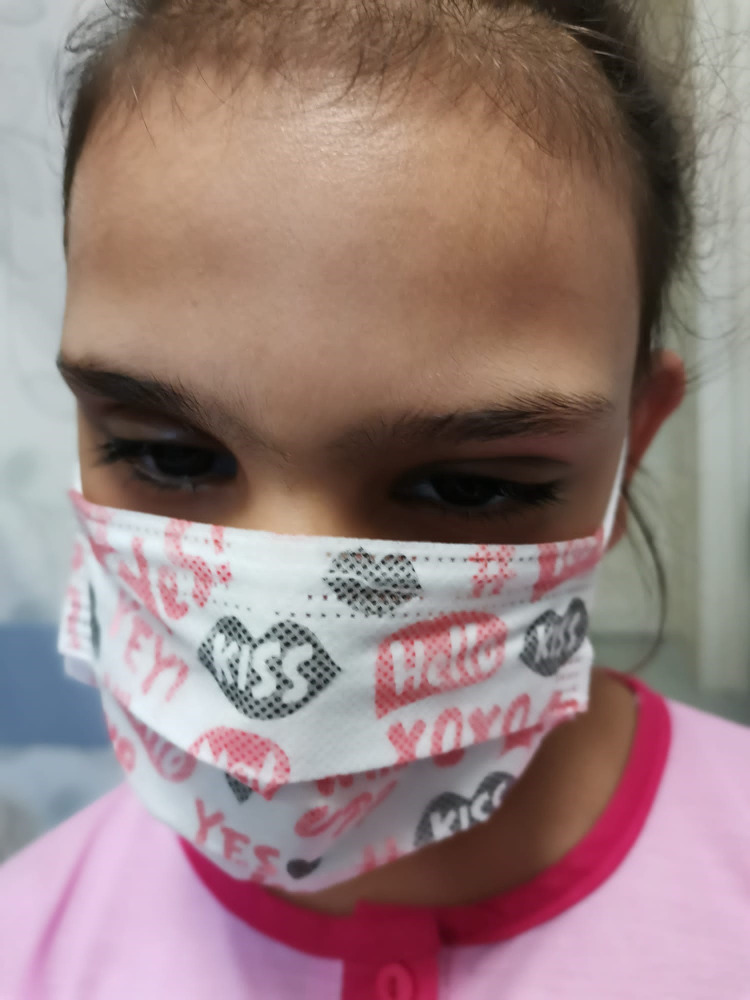
Left ocular torticollis - aphakic left eye, right ocular prothesis, head tilt to the left

We considered that the left-sided head tilt was an adaptive ocular torticollis that made the patient able to gain a free para-leukoma visual axis, allowing the optic nerve to mature to a certain level (**Fig. 7**). Scoliosis could either be due to ocular torticollis or an abnormal skeletal maturation on account of maldevelopment of neural crests. 

Rehabilitation required long follow-up with periodic measurement of IOP to detect glaucoma at an early stage. Optical correction of aphakia and amblyopia therapy are mandatory when collaboration allows. 

## Discussions

PA is an anterior segment dysgenesis that accounts for the highest type of abnormality in the “corneal congenital opacities”. In terms of treatment, there is no definitive curative procedure, while timing and surgical approach remain a debatable topic. 

Patients with type I anomaly should undergo penetrating keratoplasty (PKP) or optical-sector iridectomy within the first year of life [**8**], whereas patients with type II need a constellation of procedures, including adhesiolysis, lens aspiration, vitrectomy, glaucoma surgery (trabeculectomy, glaucoma drainage device, laser cycloablation), which deteriorates the postoperative success of a corneal transplant. There is no “gold-standard” procedure, yet most authors recommend PKP. 

In his study, Zaidman evaluated the visual prognosis of 24 patients with Peters anomaly type I after corneal transplantation. He found that 83% of children operated in their first year and 90% of children over 3 years old have clear corneas over a 78.9-month follow-up [**13**]. However, graft rejection was noted in 17% of cases and 50% were treated for glaucoma [**13**]. In a Korean study based on 19 PKP performed in children over 1 year with Peters anomaly, graft failure ranges from 30% at 1 year, 39% at 3 years, 70% at 5 years and 77% at 10 years [**18**]. Another retrospective case series that follows the outcomes of children with PK, stated the development of glaucoma and the necessity of multiple surgical procedures as independent factors for graft loss [**19**]. In addition to high risk of graft failure, pediatric PKP is a complex procedure that requires frequent examinations under general anesthesia and chronic topical steroid use, thus leading to potential complications in the long run. 

Optical iridectomy was proposed as an alternative procedure in order to obtain a free visual axis if a clear zone in the cornea is present. Oriel Spierer published the largest series of 29 optical iridectomies for Peters anomaly type I in children under 8 years old, demonstrating the success of the procedure in obtaining visual improvement in 72.4% over 42.8 months, particularly in bilateral cases [**15**]. Yet, the utility of optical iridectomy in unilateral cases needs further studies [**15**]. 

Soh and colleagues described a new technique in a case of type I PA that involved selective endothelial removal, while preserving intact DM. Visual acuity improved to 20/ 30 without the need of further corneal transplantation [**16**]. 

However, the kerato-lenticular adhesions and corneal opacity in type II anomaly make the surgical approach more difficult. To the best of our knowledge, the literature lacks surgical options for type II PA. In our case, we approached a combination of techniques including adhesiolysis, intra-lenticular lens aspiration and anterior vitrectomy. Since the whole procedure was performed in the bag, we reduced the risk of complications such as vitreous prolapse, lens matter drop, or significant post-surgery astigmatism. The cornea remained clear postoperative, except for the previous leukoma, offering a free paracentral visual axis. The complexity and uniqueness of the case relied on the difficult approach made by the cloudy cornea and dislocation of the lens in the anterior chamber, which might further increase the loss of endothelial cells. We believed that the careful manipulation and a good quality of the residual endothelial cells prevented the extension of the leukoma. No glaucoma treatment was needed for the time being on account of normal intraocular pression at multiple visits. Further follow-up was required to assess if a PKP or an IOL suturing was going to improve the visual prognosis in the future.

We found another case of type II PA using the same approach of cataract surgery without PKP, but the corneal defect was smaller, and lens was not displaced, allowing for a proper capsulorhexis. Visual acuity and follow-up results were similar to our case [**14**].

## Conclusion

To conclude, type II PA represents a very rare and complex pathology, which affects multiple corneal layers. As far as we know, this is the first case report describing cataract surgery with intra-lenticular lens aspiration and no penetrating keratoplasty in a child with dislocated lens. However, the surgical indication of our procedure is restrained to type II Peters anomaly that allows for a clear and healthy peripheral cornea associated with severe amblyopia that will lower the advantages of other procedures. In this case, lens dislocation and potential amblyopia overcome the benefits of PKP. Further interventions such as secondary IOL implantation will be taken into consideration after six-month and one-year postoperative follow-up. Additional research is needed before this surgical approach can be used in a standard manner.

**Conflict of Interest Statement**

The authors state no conflict of interest. 

**Informed Consent and Human and Animal Rights statement**

Informed consent has been obtained from the legal guardian of the patient included in the study.

**Authorization for the use of human subjects**

Ethical approval: The research related to human use complies with all the relevant national regulations, institutional policies, it is in accordance with the tenets of the Helsinki Declaration and has been approved by the review board of Clinical Emergency Eye Hospital Bucharest, Romania. 

**Acknowledgements**

None. 

**Sources of Funding**

None. 

**Disclosures**

None. 
